# Genetic Mechanisms of Migraine: Insights from Monogenic Migraine Mutations

**DOI:** 10.3390/ijms241612697

**Published:** 2023-08-11

**Authors:** Helin Gosalia, Nazia Karsan, Peter J. Goadsby

**Affiliations:** 1 Headache Group, The Wolfson Sensory, Pain and Rehabilitation Centre, NIHR King’s Clinical Research Facility, & SLaM Biomedical Research Centre, Institute of Psychiatry, Psychology and Neuroscience, King’s College London, London SE5 9PJ, UK; helin.1.gosalia@kcl.ac.uk (H.G.); nazia.karsan@kcl.ac.uk (N.K.); 2 Department of Neurology, University of California, Los Angeles, CA 90095, USA

**Keywords:** genetics, monogenic models, familial hemiplegic migraine, FASPS, sporadic hemiplegic migraine, cortical spreading depression, aura, TRESK

## Abstract

Migraine is a disabling neurological disorder burdening patients globally. Through the increasing development of preclinical and clinical experimental migraine models, advancing appreciation of the extended clinical phenotype, and functional neuroimaging studies, we can further our understanding of the neurobiological basis of this highly disabling condition. Despite increasing understanding of the molecular and chemical architecture of migraine mechanisms, many areas require further investigation. Research over the last three decades has suggested that migraine has a strong genetic basis, based on the positive family history in most patients, and this has steered exploration into possibly implicated genes. In recent times, human genome-wide association studies and rodent genetic migraine models have facilitated our understanding, but most migraine seems polygenic, with the monogenic migraine mutations being considerably rarer, so further large-scale studies are required to elucidate fully the genetic underpinnings of migraine and the translation of these to clinical practice. The monogenic migraine mutations cause severe aura phenotypes, amongst other symptoms, and offer valuable insights into the biology of aura and the relationship between migraine and other conditions, such as vascular disease and sleep disorders. This review will provide an outlook of what is known about some monogenic migraine mutations, including familial hemiplegic migraine, familial advanced sleep-phase syndrome, and cerebral autosomal dominant arteriopathy with subcortical infarcts and leukoencephalopathy.

## 1. Introduction

Migraine is a severely disabling common neurological disorder involving a range of brain regions, such as the brainstem and hypothalamus [1,2]. Over the last few decades, through human imaging studies [3,4] and preclinical and clinical experimental migraine models [5], our understanding of migraine pathophysiology has evolved, including the likely molecular mechanisms involved and therefore the consideration of novel therapeutic targets [6,7]. Broadly, migraine is classified as having two dominant clinical phenotypes, migraine without aura and migraine with aura, affecting roughly 70% and 30%, respectively [8,9]. Studies dating from the 1990s revealed that there is a genetic foundation to migraine biology, particularly through the outcomes of twin studies suggesting familial aggregation [10,11,12,13]. Further to this, migraine heritability was found to be around 42% [14], and those with migraine with aura display stronger heritability [15].

Whilst in terms of headache and other symptom phenotypes, migraine with and without aura are phenotypically similar, there may be important differences between the two subtypes biologically, which remain poorly understood. Migraine aura has been very challenging to study in humans due to logistical issues with ictal capture: it most often precedes headache and can be brief and less than an hour in duration, and it is uncommonly triggered by natural provocation factors that patients report are reliable triggers for them [16]. Interestingly, human experimental migraine models, when provocation agents are used to induce migraine-like attacks, generally do not provoke aura [17], and those that have been tested in familial hemiplegic migraine (FHM) patients do not provoke FHM attacks [18,19,20].

Gaining insights into the genetic architecture of migraine, particularly through the monogenic mutations, is important in order to understand better the mechanisms involved in aura overall and in the severe aura phenotypes associated with some of these conditions and to increase treatment targets. It is also important to understand the differences, if any, in migraine susceptibility and pain processing mechanisms between migraine with and without aura. Large case-control genome-wide association studies (GWAS) from different centres have successfully identified several indicative susceptibility loci in patients with migraine in general, confirming vascular and neuronal bases to migraine, but the results have thus far failed to advance migraine therapeutics and lead to a change in patient management. Further work to identify causal polymorphisms and their effects on migraine mechanisms via transcriptomics and functional identification, perform gene expression studies, and understand the interaction between genetic and epigenetic factors in migraine will be important in the future to allow the data from GWAS studies to be translated to patient outcomes and treatment [21,22,23,24]. Migraine is typically associated with a polygenic inheritance based on these studies, whereby the underlying risk is influenced by several gene variations [25]. Less common, but nonetheless important, is monogenic migraine, which includes migraine with aura as part of the clinical phenotype. Familial hemiplegic migraine is a rare monogenic migraine form [25], described as migraine with aura involving motor weakness on one side of the body [9]. Hemiplegic migraine can be divided into familial hemiplegic migraine (FHM), the monogenic form that can be caused by mutations in any of three identified causative genes, and sporadic hemiplegic migraine (SHM). FHM fits the criteria for hemiplegic migraine accompanied by at least one first or second degree relative affected. Comparatively, should a patient have no family migraine history or an identifiable FHM gene and fit the criteria for hemiplegic migraine, they would be diagnosed as SHM [9]. Importantly, up to a quarter of patients with a positive family history lack an identified mutation in any of the three named genes, suggesting additional, perhaps yet to be recognised, genes are also involved [26]. The scope of ion channel gene mutations that may cause migraine with aura phenotypes is increasing [27], and further causative genes may be identified in due course. See Table 1.

Other monogenic mutations can cause migraine with aura of the non-hemiplegic type. These include mutations in the TWIK-related spinal cord potassium channel (TRESK) gene, which alters gene function, which in turn can affect nociceptive processing, and familial advanced sleep phase disorder (FASPS), caused by mutations in casein kinase 1δ (*CK1δ*), leading to disrupted circadian rhythms and migraine with aura [28,29]. Other monogenic vascular disorders can feature migraine in the clinical phenotype and include cerebral autosomal dominant arteriopathy with subcortical infarcts and leukoencephalopathy (CADASIL), caused by mutations in the NOTCH3 gene, resulting in subcortical infarcts, cognitive decline, and migraine with aura [30]. Migraine with aura can precede the diagnosis and other clinical features by decades. These mutations and conditions provide feasible links biologically to account for the associations between migraine and vascular disease and circadian dysfunction, alluding to the likely important roles of the cerebral vasculature and brain areas such as the hypothalamus, which is involved in homeostatic regulation, in migraine mechanisms.

**Figure 1:**
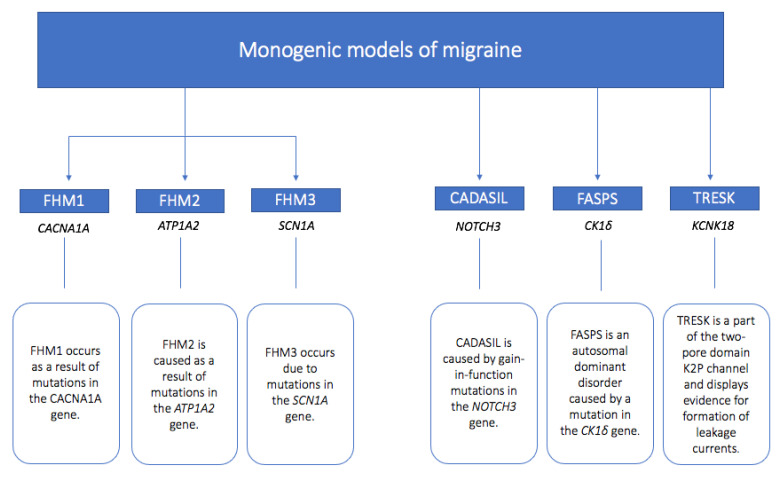
Summary of monogenic models of migraine with the relevant genes involved and implicated mutation summary.

This review will provide a concise insight into the genetics of migraine, detailing the developments of experimental monogenic models of migraine. We will feature the common monogenic migraine mutations, with specific reference to causative mutations causing familial hemiplegic migraine, and also include those causing migraine with other aura subtypes and with other symptoms, as well as CADASIL as a vascular disorder with which migraine with aura is associated. We will dissect findings from animal studies and the human clinical phenotypes described to give an overview of what has been learnt about cortical spreading depression (CSD) and aura mechanisms, as well as overall migraine biology, comorbidities, and associations with other physiological processes through the monogenic representations of migraine. We will start with an overview of the current understanding of the biological basis of migraine aura, as this will provide a background for understanding the insights from the monogenic migraine models. A summary of the discussed mutations is provided in Figure 1.

## 2. CSD, Genetics, and Animal Models

CSD was first identified in 1944 [31] through Leão’s thesis investigation of experimental epilepsy. Through his studies, Leão marked that following electrical or mechanical stimulation of the cerebral cortex, there was depression of EEG activity [32]. CSD was therefore described as an electrophysiological phenomenon and is now recognised as a wave of neuronal depolarisation succeeded by suppressed neural activity propagating through the cerebral cortex [1,33,34,35]. This self-propagating wave of depolarisation followed by hyperpolarisation is thought to disturb typical neuronal functioning and is assumed to be the neurophysiological correlate to migraine aura [36]. This theory has been supported by human functional imaging work [37,38,39] during aura. Whilst CSD can be provoked in animal models, through cortical injury, electrical stimulation, or potassium chloride application to the cortex, it can occur spontaneously in humans. Membrane ionic changes occur, such as increased extracellular potassium and decreased sodium, chloride, and calcium concentrations [40]. CSD also causes release of neurotransmitters, such as glutamate and aspartate, during depolarisation [41]. Neuronal depolarisation removes the voltage-sensitive Mg^2+^ block of the *N*-methyl-D-aspartate (NMDA) receptor and sensitises the receptor to small increases of interstitial glutamate [42]. Glutamate, via the NMDA receptor, promotes further K^+^ and glutamate release and ongoing neuronal depolarisation to facilitate CSD repetition and spread [43]. Cortical blood flow alterations occur as a consequence of CSD, and brief vasoconstriction during depolarisation is followed by vasodilatation and increased blood flow for 1–2 min, and then a further reduction in cerebral blood flow, which is more persistent in CSD-affected brain regions [44,45].

The exact cause of CSD and the association with trigeminovascular nociception in migraine are yet to be fully elucidated, however, genetic factors likely contribute to CSD predisposition [46,47]. Certainly, those with monogenic migraine mutations have an increased propensity to spontaneous and triggered aura, such as after mild head injury [48], and animal models suggest an increased tendency for repetitive CSDs and increased CSD propagation velocity [49].

Over the years, a range of clinical, preclinical, and neuroimaging studies have suggested that CSD has a focal role in migraine with aura pathophysiology [50]. Migraine genetics has contributed to this significantly, namely because the monogenic migraine mutations, in particular the FHM ones, can produce such florid aura phenotypes, and modeling of these experimentally has allowed further investigation of CSD mechanisms. Rodent genetic models deliver insights into the propensity for CSD and the cellular and mechanistic changes associated with CSD [51]. Many, but not all, migraine drugs used in clinical practice alter CSD thresholds, but these also generally have a broad range of central nervous system effects [52]. To date, there has been little evidence to suggest that some treatments are more efficacious than others for clinical aura and for the more florid aura phenotypes. Flunarizine has some evidence for efficacy in aura [53] and is widely used in Europe, but it is not licensed in many parts of the world, including the United Kingdom and United States. Understanding CSD mechanisms and their interaction, if any, with nociceptive mechanisms and the identification of potential therapeutic substrates within the monogenic migraine models would be a valuable strategy going forward.

## 3. Monogenic Models of Migraine

### 3.1. Familial Hemiplegic Migraine Type 1 (FHM1)

As well as causing familial hemiplegic migraine [54,55], FHM mutations can also cause additional clinical syndromes, with other transient symptoms such as seizures or cerebral oedema [56], or fixed signs such as nystagmus and ataxia [57].

The genetic basis of FHM was first identified via mutations in the *CACNA1A* gene in multiple FHM family pedigrees [54], and mutations in this gene now represent the clinical syndrome of FHM1 [54]. The gene encodes the pore-forming α-1 subunit of the neuronal voltage-gated calcium channel Ca_V_2.1 (or P/Q-type Ca^2+^ channel), located presynaptically in brain and cerebellar neurons, with an important role in controlling neurotransmitter release [58,59]. To date, known FHM1 mutations are inherited in an autosomal dominant fashion, and they typically cause gain-of-function effects on human P/Q-type Ca^2+^ channels [60,61], causing calcium influx into neurons, enhanced glutamatergic neurotransmission, cortical hyperexcitability, and, therefore, increased susceptibility to CSD [62,63]. Differences in symptoms and symptom severity can be observed across the different variants of FHM1 [64].

Several transgenic mouse models for FHM1 via knock-in mice have been produced and have demonstrated that the FHM1 mutations cause gain-of-function effects and, overall, an increased susceptibility to CSD [65], but also that different gene mutations cause differing animal phenotypes and symptom severities. Sensory hyperexcitability of trigeminal ganglion neurons has also been demonstrated in these mice [66,67], as well as sensitivity to provoked head pain and light aversion [68] and cortical anoxia related to prolonged CSD [69]. Interestingly, within these mouse models, female sex hormones increased CSD susceptibility [70], and androgens play a reverse role [71]. These data suggest that other factors such as hormones may alter the clinical phenotype and migraine threshold in FHM and that male–female differences may account for some phenotypic diversity in the disease. Supporting this, even within the same family affected by FHM1 with the same missense gene mutation, the FHM1 clinical phenotype can vary significantly, suggesting that other genetic, environmental, and hormonal factors may be involved in the interictal migraine threshold, the clinical display of symptoms by different family members [72], and the variable penetrance of the mutations.

To date, there have been two reported FHM1 knock-in mouse models carrying gain-of-function mutations, R192Q and S218L, in the *CACNA1A* gene [49,65]. The R192Q mutation in animals results in a milder phenotype [65], while mice with the S218L mutation exhibit a pronounced phenotype characterised by cerebellar ataxia and seizures; these manifestations are consistent with the clinical phenotype observed in some individuals with FHM1 mutations [49]. Studies conducted using FHM1 knock-in mice have revealed several significant findings. In addition to an increased susceptibility to CSD, these mice also exhibit alterations in the balance between neuronal excitation and inhibition [62,65,70]. FHM1 mutants seem to display prolonged hemiplegia following a single induced CSD, and this CSD spread [73] from cortex to striatum, whereas this did not occur in wild-type mice and may explain the more severe hemiplegia in the FHM1-affected patients. In addition, the cortical oligaemia following CSD was more prolonged in this model, with a larger increase in intracellular calcium and reduced tissue oxygenation [69]. More severe FHM1 mutations were associated with seizures following CSD and an increased risk of CSF propagating to subcortical structures, correlating with the more severe clinical phenotypes in some FHM1 syndromes [70].

Within these models, enhanced glutamatergic synaptic transmission increases cortical hyperexcitability via pyramidal cells and fast-spiking interneurons, whilst GABAergic neurotransmission remains unaltered in FHM1 mice [62]. These inhibitory–excitatory modulating cortical circuits control gating of sensory information [74], and defects in these in FHM1 cause cortical electrolyte imbalance. The spread of CSD from cortical to subcortical structures in FHM1 mice can be inhibited by pregabalin, which supports the fact that CSD initiation and spread are likely linked to excitatory neurotransmission [75] in FHM1, related to increased synaptic glutamate release. Interestingly, and contrary to this finding, pregabalin has not emerged as useful a clinical treatment of hemiplegic migraine. The involvement of the implicated calcium channel in various parts of the trigeminovascular system’s neurotransmitter pathways suggests these mutations may have roles in other aspects of migraine, and this area deserves further study. Calcitonin gene-related peptide (CGRP) release has not been shown to be altered in CSD-provoked or basal dural afferent activation in FHM1 mice, and, paradoxically, capsaicin and CGRP were shown to reduce vasodilatation in these mice, suggesting that CGRP may play an alternative, if any, role in FHM compared to more common migraine [76,77]. Dural electrical stimulation does not increase neuronal activity in the trigeminocervical complex (TCC) in FHM1 mice, although changes in thalamic processing, specifically in the posterior and centromedian nuclei, can be observed. This suggests that trigeminal ganglion afferents may not be affected in FHM1, but central trigeminothalamic neurons are [76].

Episodic ataxia type 2 and spinocerebellar ataxia type 6: Other mutations in *CACNA1A* can cause episodic ataxia type 2 and spinocerebellar ataxia type 6, two ataxic disorders also associated with migraine as part of the clinical phenotype in many of those affected [78]. Episodic ataxia type 2 mutations typically cause a loss-of-function with decreased intracellular calcium influx [78], whilst spinocerebellar type 6 mutations cause a gain-of-function [79]. Both of these disorders are associated with a higher rate of migraine and nausea, and the penetrance and clinical expression of the mutations are highly variable, with several different mutations causing each syndrome identified thus far. The variable clinical expression of different mutations within the *CACNA1A* gene, the phenotypic heterogeneity amongst those affected by the same genetic mutation, the sometimes overlapping clinical phenotypes, and the high prevalence of migraine amongst all the mutations is interesting and suggests shared biological mechanisms between ion channel-mediated alterations in neurotransmission and cortical excitability in migraine and these other neurological conditions [80,81].

### 3.2. Familial Hemiplegic Migraine Type 2 (FHM2)

FHM2 is caused as a result of mutations in the *ATP1A2* gene, first identified in 2003 [55]. The *ATP1A2* gene encodes the α-2 subunit of the Na^+^,K^+^-ATPase [55]. Whilst *CACNA1A* mutations in migraine exhibit gain-of-function effects, *ATP1A2* mutations produce loss-of-function of sodium–potassium ATPases and, in turn, rising K^+^ levels within the synaptic cleft, thus altering cell membrane sodium gradients in key astrocytic cells and affecting glutamatergic neurotransmission, as well as reducing extracellular potassium clearance [55]. Mutations in *ATP1A2* lead to disruptions in ion homeostasis and neuronal excitability [82,83], contributing to the development and onset of migraine attacks. This can occur via increased or decreased potassium clearance, reduction in the sodium/potassium turnover rate [84], or functional inactivation by impaired protein stability [85]. Again, inheritance is usually autosomal dominant and produces a florid and heterogeneous clinical phenotype in addition to migraine with hemiplegic aura due to more than 80 causal variants in the gene being identified and de novo mutations being common [86]. The phenotype can include recurrent coma, fever, and hypokalaemic periodic paralysis [87]. Variants in *ATP1A2* have been most commonly identified in those diagnosed with SHM and may lead to SHM becoming identified as FHM because of a de novo mutation, which can then be passed down [88].

Interestingly, family members experiencing FHM2 often have a medical history of seizures and diagnosed epilepsy [55,89,90,91]. Given the shared mechanisms between migraine and epilepsy, both episodic disorders of the brain and the ability of one to trigger the other [92], Deprez and colleagues studied whether mutations in *ATP1A2* are common for both migraine patients and epilepsy patients [93]. They conducted a mutation analysis of *AT1PA2*; the results revealed that two Belgian families had two novel *ATP1A2* mutations, and both of these mutations occurred in families who experienced migraine and epilepsy. The findings suggested that it is important to take a history of epilepsy in migraine patients and vice versa. This gene should be studied in patients presenting with both disorders or each one individually. Again, the heterogenous phenotype and effect of the ion channel mutations in migraine can offer insights into migraine comorbidity with other related disorders of cortical excitability, such as epilepsy.

FHM2 knockout mice have generated interesting insights into the functional relevance of these mutations. Homozygous *ATP1A2* knockout mice do not survive beyond birth due to congenital neurodegeneration [94], and significant neurological malformations and neonatal death have been reported in humans who have biallelic loss-of-function mutations in *ATP1A2* [95]. Heterozygotic mice have a propensity to CSD and delayed recovery from it [96,97], as well as abnormal behaviour and neurological defects, thought to be secondary to reduced glutamate and potassium astrocytic clearance [98]. Na^+^,K^+^-ATPase pump activity changes, and therefore increased extracellular potassium and glutamate levels lead to altered cortical excitability. Contrary to FHM1 knock-in mice, cerebral blood flow following CSD was similar in FHM2 knockout mice and in wild-type mice, but the recovery to baseline spontaneous activity following CSD was slower [99]. Interestingly, a particular knockout α2-Na/K ATPase mouse model exhibited transient motor paralysis and spontaneous CSDs [100], supporting the clinical phenotype of spontaneous aura and hemiplegia observed in humans with FHM.

FHM2 knock-in mouse models have been somewhat less studied than FHM1 knock-in models. Notably, Bottegar and colleagues used an α-2 subunit of the Na^+^,K^+^-ATPase gene knock-in mouse model (α_2_^+/G301R^), identified through the FHM2-related *G310R* mutation found in two families [99]. The researchers focused on gaining an understanding on how this mutation in the FHM2 mouse model affects the glutamate system, which is fundamental for synaptic transmission. Several findings are drawn from this interesting research. Firstly, via an alteration in glutamate release and reuptake mechanisms, increased levels of glutamate in lysates/cells from a number of areas in the brain were observed when compared to the wild-type mice [99]. Secondly, the knock-in mice exhibited various behavioural changes, such as compulsive behaviour, behaviours modelling obsessive-compulsive disorder, a reduction in sociability, and depression-like manifestations when exposed to stress. This is a particularly interesting finding given the demonstration that the α_2_^+/G301R^ mice demonstrated social and characteristic deviations mirroring those that occur clinically in patients with migraine [101]. The research detailed that the observed changes are probably attributable to a reduction in glutamate clearance from the synaptic cleft, particularly in the female mice due to the consequences of female sex hormones and associated cycles [99]. The translatable findings from this preclinical study to observations made clinically make a scientifically positive overture and may result in feasible avenues of therapeutic targets to be developed for specific monogenic migraine subtypes.

FHM2 mice help to support a glutamatergic theory for the propensity to CSD mechanisms [98,99]. FHM2 mutants display reduced glutamate clearance at synapses of cortical astrocytes and reduced glutamate transporter expression at synapses [98]. Compulsive behaviour displayed by FHM2 mutant mice can be reversed by memantine, an NMDA receptor antagonist, which reduces glutamatergic neurotransmission [99]. There is clinical evidence of memantine efficacy in migraine [102,103,104]. Again, altered cortical circuit function and altered electrolyte balance are likely implicated, as in FHM1, related to reduced potassium and glutamate clearance.

### 3.3. Familial Hemiplegic Migraine Type 3 (FHM3)

Mutations in the *SCN1A* gene cause FHM3, identified most recently in 2005 [105]. FHM3 is the rarest of the FHM subtypes. The *SCN1A* gene encodes the α-1 subunit of the voltage-gated Na^+^ channel Na_V_1.1, which controls the sodium permeability of GABAergic interneurons within the central nervous system [106]. Similarly to FHM2, epilepsy with *SCN1A* mutations is very common [107]. SCN1A mutations can cause Dravet syndrome, a rare childhood epileptic syndrome [108]. Epilepsy can be comorbid with migraine with hemiplegic aura among those affected by FHM3 [109].

Whilst epilepsy mutations in the gene are usually loss-of-function and lead to reduced inhibitory interneuron firing, increased selective cortical interneuron firing, and therefore seizures [110], in FHM3, the gene mutations in migraine tend to cause gain-of-function and cause increased GABAergic firing, increased extracellular potassium and glutamate, and an increased propensity to CSD [111]. Mixed loss- and gain-of-function mutations causing both FHM3 and epilepsy have been reported in one family [112]. Knockout mice for *SCN1A* have epilepsy and ataxia [113].

In 2020, data on the first FHM3 knock-in mouse model were published [114]. With the first FHM3 transgenic mouse model, this study demonstrated spontaneous cortical spreading depolarisation in animals. CRISPR/Cas9, a genome editing method, was adopted to introduce the mutation into the endogenous *SCN1A* gene. The mice demonstrated a low CSD induction threshold and a lower electrical threshold for CSD. According to the findings and conclusions, *SCN1A*^L263V^-expressing mice displayed spontaneous CSDs spreading from the visual cortex to the motor cortex. The results translate to our current understanding of visual aura features from neuroimaging studies of patients with migraine with aura [3,38,115], which suggests an occipital and visual cortex onset, with subsequent spread to other cortical areas, such as those controlling sensation or speech. It is of note that, rather unexpectedly, all of the heterogenous *SCN1A*^L263V^ died. The results further indicate the role of GABAergic interneuron hyperactivity as a mechanism for CSD. Desroches and colleagues’ findings align with this preclinical model [116]. This study hypothesised that hyperexcitability of GABAergic interneurons can essentially ignite CSD through a loss of inhibition. Furthermore, supportive studies have shown that hyperexcitability of these GABAergic interneurons does indeed act as mechanism to trigger CSD [117,118]. However, further animal models of FHM3 are required to draw robust conclusions and to further add to our understanding of the mechanisms involved in this rare FHM subtype.

### 3.4. Cerebral Autosomal Dominant Arteriopathy with Subcortical Infarcts and Leukoencephalopathy (CADASIL)

Cerebral autosomal dominant arteriopathy with subcortical infarcts and leukoencephalopathy (CADASIL) is caused by gain-of-function mutations in the *NOTCH3* gene, located on chromosome 19q13 [30,119]. It is noteworthy that headache attributed to CADASIL resembles migraine with aura phenotypically, but exhibits an unusual high frequency of aura and has its own separate definition aside from migraine with aura within the International Classification of Headache Disorders, third edition [9]. Moreover, despite a common clinical migraine with aura phenotype, there are likely shared yet distinct mechanisms in CADASIL relative to migraine with aura in general. The *NOTCH3* gene encodes a transmembrane receptor expressed in smooth muscle cells and pericytes of small vessels in humans [120,121]. In CADASIL, the typical mutation implicates the expansion or duplication of a specific sequence of six amino acids within the EGFr domain [122]. This genetic modification results in atypical aggregation of the mutant *NOTCH3* protein in the walls of the small blood vessels [123], causing a syndrome of recurrent subcortical brain ischaemia and death. Inheritance is usually autosomal dominant. Approximately 75% of patients with CADASIL experience migraine attacks, often migraine with aura, and migraine with aura can precede the CADASIL diagnosis by some years [124,125,126]. The mechanism through which there is an increase in aura prevalence in patients with CADASIL is unknown. However, Liem and colleagues suggested that this could be due to a myriad of factors such as the *NOTCH3* gene acting as a migraine aura susceptibility gene or an increased susceptibility to CSD [126]. Interestingly, a study by Oka and colleagues deduced that enlarged focal cerebral ischemic infarcts in mutant CADASIL result in increased susceptibility to CSD. Although this was not strictly a migraine study, the involved gene mutations mirror existing knowledge about CADASIL and CSD, further strengthening this phenomenon [127]. However, further research to characterise the association between the NOTCH3 gene and aura mechanisms is required. Migraine with aura has been associated with white matter intensities, but these are subclinical and are not clearly associated with cognitive dysfunction [128,129]. There may well therefore be some mechanisms in vascular walls that are shared between CADASIL and non-monogenic migraine with aura, but this area needs further work.

### 3.5. Familial Advanced Sleep-Phase Syndrome (FASPS)

Familial advanced sleep-phase syndrome (FASPS) is an autosomal dominant disorder that leads to a tendency to wake up early in the morning and sleep early at night due to the shift of the circadian cycle and rhythm caused by a mutation in the *CK1δ* gene [29,130]. This gene encodes casein kinase 1δ [28]. *CKIδ* holds an integral role in the regulation of circadian rhythm [131], which is a natural 24 h oscillation that regulates the sleep–wake cycle and other homeostatic functions [132]. This missense mutation has been connected to migraine, with affected patients showing disrupted circadian rhythms and migraine with aura [1]. An intriguing investigation by Brennan and colleagues noted two distinct missense mutations at T44A and H46R in two families who presented with migraine with aura and FASPS [28]. In mice with the *CK1δ*-T44A mutation, it was observed that the threshold for CSD was lower, further suggesting a possible role of this gene mutation in the underlying biology of migraine with aura. These mice also display increased sensitivity to pain following administration of NTG, a potent experimental migraine trigger, in terms of reduced heat and mechanical withdrawal thresholds in mutant mice, and increased immunoreactivity in the trigeminal nucleus caudalis (TNC) following NTG administration in the mutant mice compared to wild-type [28]. There has been an interest in the interplay between sleep and migraine mechanisms over many years, given their bidirectional association [133], and the FASPS model provides novel insights into the relationship between circadian rhythms (including those affecting sleep and feeding) and migraine, in particular with regards to the involvement of the hypothalamus and hypothalamic neurotransmitters as possible novel therapeutic targets in migraine.

### 3.6. TWIK-Related Spinal Cord Potassium Channel (TRESK)

The TWIK-related spinal cord potassium channel is a part of the two-pore domain K2P channel and displays evidence for the formation of leakage currents with enhanced expression in sensory ganglia, supporting a role in pain processing [1,134,135,136]. There has been evidence that knockout TRESK mice display hyperexcitability of the sensory ganglia and trigeminal hyperexcitability [137], with behaviour consistent with pain [136,138], whilst another TRESK mutation also affecting sensory neurons does not cause the same trigeminal hyperexcitability [139]. These divergent effects of mutations on the same gene have been suggested to be due to altered gene function from frameshift mutations [140].

Frameshift mutations, such as F139WfsX24, in the *KCNK18* gene coding for TRESK induce a complete loss-of-function in a particular family suffering with migraine with aura [141]. A more recent study using the genome editing technique CRISPR-Cas9 was adopted to correct the F139WfsX24 mutation and display a reversal of the neuronal hyperexcitability, which linked the clinical phenotype to the mutation and further strengthened the concept that TRESK plays a role in migraine and aura mechanisms [134]. Interestingly, as this genome editing technique was able to reverse neuronal hyperexcitability, the design of TRESK-targeted therapeutics aiming to do the same may form an exciting novel therapeutic opportunity in the future.

When TRESK and two other K2P channels are knocked out in a mouse model (TREK1 and TREK2), the mice display a craniofacial allodynic phenotype. TRESK-mediated TREK1 and 2 downregulation is felt to contribute to migraine susceptibility [140]. These models support a role of TRESK and TREK channels in migraine mechanisms.

## 4. Insights and Future Directions

Whilst monogenic migraine mutations are considerably rarer than the polygenic more common migraine, they offer valuable insights into aura physiology and the shared underpinnings of migraine with other neurological disorders, as well as with possible shared mechanisms with vascular disorders. The majority of the mutations are ion channel mutations, and the FHM disorders can be classified as channelopathies [142]. A change in gene function alters synaptic neurotransmission and neuronal excitability via glutamatergic mechanisms and altered cortical excitation/inhibition balance. This contributes to disordered sensory gating and synaptic plasticity and changes the propensity to CSD (enabling spontaneous CSD to occur in the cortex of those affected) and delays cortical recovery from CSD in the FHM rodent models. These responses may be further modulated by other factors such as gender, hormonal differences, and stress. Whilst it is difficult to translate the findings of these to more common migraine, these models do offer interesting insights into the differences in migraine amongst different migraine subtypes, the potential for glutamatergic treatment in migraine with aura, which has some clinical support [143], and potential prediction of treatment response between therapies in these patient groups. Despite a lack of systematic evidence, there is the suggestion that targeting CGRP may not be efficacious in this patient group, but real-world longitudinal evidence from those treated with CGRP-targeted treatments will reveal more about this in due course. Targeting glutamate via NMDA receptor antagonism [144] or mGluR5 antagonism [145] on the other hand may pose potential therapeutic options for these patients, and further large-scale systematic therapeutic studies are warranted. Other genetic migraine mutations such as CADASIL and FASPS provide feasible biological and physiological links between migraine and other comorbid disorders and contribute to the GWAS findings of likely implicated vascular and neural genetic mechanisms in migraine. Specific targeting of hypothalamic neurotransmitters, including those involved in circadian rhythms such as orexins [146], have thus far not shown promise in migraine therapeutics, but more focused targeting of individual receptor subtypes, such as neuropeptide Y Y1 agonism (a hypothalamic neuropeptide involved in the regulation of feeding and sleep via interaction with the orexins) may hold potential in the future [147,148].

## 5. Conclusions

Genes such as *CACNA1A*, *ATP1A2*, and *SCN1A* have played an instrumental role in shaping the genetic understanding of CSD and aura mechanisms and therefore migraine susceptibility. Animal models and detailed genetic studies in affected families have focused our attention on the cellular and molecular mechanisms of migraine biology, such as neuronal hyperexcitability and CSD and the role of neurotransmitters and ion channels, carving the likely neurochemical foundations of migraine biology. Rodent models allow us to gain a translational understanding of the monogenic mutations, which, from bench to bedside, may allow translation to the clinical world to ideally improve targeted, specific, and evidence-based management of patients with monogenic migraine. Given patient-facing research and clinical understanding can somewhat limit our scope to extrapolate research to understand the biology in depth, particularly in these rare and phenotypically heterogenous genetic disorders, monogenic animal models have formed the basis to deepening the understating of aura pathophysiology. It would be of value for preclinical studies in this area to be developed further to systematically test drug agents within these rodent models to hopefully in the future lead to the availability of targeted therapies for this particularly disabled patient population. To understand better the complex mechanistic underpinnings of migraine biology overall, genome-wide association studies and other large-scale genetic studies are important for detecting further migraine susceptibility loci and, in turn, understanding how these loci may be involved in migraine and if any have the potential to become therapeutic targets. A very understated and perhaps underutilised tool we have is our patients and their clinical phenotypic data, which, when acquired prospectively and systematically, could help us further understand the association between migraine and other neurological and vascular disorders over time, the differing phenotypes amongst those affected by monogenic migraine, and the emergence of potentially relevant new mutations, which may become increasingly recognised in clinical practice. The spectrum of FHM mutations even in the last decade has increased, and there are new mutations being identified, which can cause the phenotype alongside other neurological disorders such as those associated with PRRT2 mutations [149]. Increasing identification of potentially implicated genes in these conditions, as well as de novo mutations, may facilitate further therapeutic studies. Ultimately, an amalgamation of our current understanding, genome-wide association studies, animal models, clinical insights, and collaborations of ideas will enhance the understanding of migraine and aura mechanisms, ultimately to treat and improve the lives of those afflicted.

## Figures and Tables

**Table 1:** Familial hemiplegic migraine types and associated features.

	FHM1	FHM2	FHM3
Genes	*CACNA1A*	*ATP1A2*	*SCN1A*
Chromosome location	19p13	1q23	2q24
Year of identification	1996	2003	2005
Mutation summary	Encodes the α-1A subunit of the P/Q type calcium channel	Encodes the α-2 subunit of the Na^+^,K^+^-ATPase	Encodes the α-1 subunit of the voltage-gated Na^+^ channel Na_V_1.1
Typical functional modulation	Gain-of-function	Loss-of-function	Gain-of-function

## Data Availability

Not applicable.
